# Effects of monoglycerides of short and medium chain fatty acids and cinnamaldehyde blend on the growth, survival, immune responses, and tolerance to hypoxic stress of Pacific white shrimp (*Litopenaeus vannamei*)

**DOI:** 10.1371/journal.pone.0308559

**Published:** 2024-08-08

**Authors:** Tirawat Rairat, Lalitphan Kitsanayanyong, Arunothai Keetanon, Putsucha Phansawat, Parattagorn Wimanhaemin, Natnicha Chongprachavat, Wiranya Suanploy, Edwin Pei Yong Chow, Niti Chuchird

**Affiliations:** 1 Faculty of Fisheries, Department of Fishery Biology, Kasetsart University, Bangkok, Thailand; 2 Faculty of Fisheries, Department of Fishery Products, Kasetsart University, Bangkok, Thailand; 3 Berg & Schmidt Asia Pte Ltd, Singapore, Singapore; National Cheng Kung University, TAIWAN

## Abstract

Free fatty acids have long been used as dietary supplements in aquaculture, but the application of monoglycerides has increased interest in more recent times. The study aimed to investigate the effects of dietary short- and medium-chain fatty acid monoglyceride and cinnamaldehyde (SMMG) on the growth performance, survival, immune responses, and tolerance to hypoxic stress of Pacific white shrimp (*Litopenaeus vannamei*). In Experiment 1, shrimp post-larvae were divided into 4 groups with 6 replicates and fed with diets supplemented with 0 (control), 0.3, 0.4, and 0.5% diet for 30 days. The final body weight and survival rate were determined. In Experiment 2, the juvenile shrimp from Experiment 1 were subjected to hypoxic stress conditions (dissolved oxygen level 2–2.5 mg/L) for 14 days, then the specific growth rate (SGR), survival rate, intestinal *Vibrio* spp. count, immune responses, and histopathological change of the hepatopancreas were analyzed. Following the 30-day feeding trial, the results revealed that the final body weight and survival of the 0.3–0.5% SMMG groups (2.81–3.06 g and 74.00–84.33%, respectively) were significantly higher than the control shrimp (1.96 g and 68.33%, respectively). In the hypoxic stress experiment, the survival rates of shrimp fed 0.4–0.5% SMMG (71.67–80.00%) were significantly higher than the control (51.67%). Although the SGR were not affected by SMMG supplementation, all immune parameters evaluated were significantly enhanced, and the intestinal *Vibrio* spp. counts were significantly decreased in the 0.4–0.5% SMMG-fed shrimp; the histopathological structure of the hepatopancreas was also improved in these shrimp compared to the control. Our findings indicated that SMMG as a feed additive has beneficial effects in improving shrimp health and increasing tolerance to hypoxic conditions.

## Introduction

Fatty acids are organic acids with aliphatic chains. Those containing fewer than 6 carbon atoms such as acetic acid (C2), propionic acid (C3), and butyric acid (C4) are classified as short-chain fatty acids (SCFA), while those with a chain length between 6–12 carbon atoms, including caproic acid (C6), caprylic acid (C8), capric acid (C10), and lauric acid (C12), are categorized as medium-chain fatty acids (MCFA) [1]. SCFA is a product of microbial fermentation of carbohydrates in the intestine [2], whereas MCFA is naturally present in milk, coconut oil, and palm kernel oil [1]. The beneficial effects of dietary organic acids in improving aquatic animal health including enhanced growth performance, immune response, and disease resistance are well-documented [2,3]. These desirable outcomes are usually attributed to the antimicrobial property of organic acids as well as their ability to improve nutrient utilization [3,4]. It has been demonstrated that caprylic acid exerts a greater inhibitory effect on the growth of *Vibrio harveyi* and *V*. *parahaemolyticus* at pH 5 than at pH 6–7 [5]. Considering that high levels of *Vibrio* spp. in the shrimp poses a significant risk of disease outbreak [6], a reduction in *Vibrio* spp. level would minimize the risk of vibriosis. Additionally, the enhancement in nutrient utilization is associated with a decreasing gut pH, which in turn enhances the digestibility of some nutrients [3].

The use of fatty acid monoglycerides, a molecule of glycerol linked to a fatty acid through an ester bond, is generally preferred over free fatty acids for several reasons. Firstly, while the free fatty acids contain an ionizable functional group (i.e.,–COOH, carboxyl group) that makes them less active against pathogenic bacteria at high pH values, monoglycerides are active across a wide pH range due to the absence of an ionizable functional group. Secondly, unlike free fatty acids, which undergo significant absorption in the upper digestive tract, monoglycerides remain intact until reaching the small intestine, where they can exert their beneficial effects. Lastly, free fatty acids often have a strong odor, which is not the case for monoglycerides [1,7–9]. Unlike free fatty acids, monoglycerides exert antimicrobial activity principally via membrane disruption due to the amphipathic property [7,10–12]. Monoglycerides form micelles at concentrations above critical micelle concentration (CMC), penetrating the cell membrane and altering membrane permeability. Typically, monoglycerides with longer chains exhibit lower CMC values and are more potent than those with shorter chains [8,9,13]. The antibacterial activity of monoglycerides is generally superior to their free fatty acid counterparts [14,15].

Among MCFA monoglycerides used as dietary supplements in animal production, one of the most popular is monolaurin or glycerol monolaurate (monoglyceride of lauric acid) [11]. The reported effective inclusion rates for growth promotion range from 0.1–0.6% diet in fish including juvenile hybrid grouper (*Epinephelus fuscoguttatus* × *Epinephelus lanceolatus*) [16], large yellow croaker (*Larimichthys crocea*) [17], Nile tilapia (*Oreochromis niloticus*) [18], and pompano (*Trachinotus ovatus*) [19], and 0.07–0.1% diet in Pacific white shrimp (*Litopenaeus vannamei*) [20,21]. In addition, investigations into the health-promoting effects of monoglyceride mixtures of SCFA and MCFA have been conducted, revealing promising results [22–24].

Cinnamaldehyde, a biologically active compound found in cinnamon barks (*Cinnamomum* spp.), is another promising feed additive. It is well-recognized for antimicrobial activity through multiple mechanisms including membrane disruption as well as inhibitions of cell division, ATPase, biofilm formation, and quorum sensing [25,26]. Other properties include anti-inflammatory, antioxidant, and cardioprotective effects [26]. Cinnamaldehyde was proven to be beneficial for the health promotion of grass carp (*Ctenopharyngodon idella*) [27], tongue sole (*Cynoglossus semilaevis*) [28], and Pacific white shrimp [29–31] when supplemented in the aquafeed at the inclusion rate of 0.02–0.1 and 0.04–0.15% diet for fish and shrimp, respectively.

Given that evidence of monoglycerides and cinnamaldehyde in promoting the good health and performance of aquatic animals is plentiful and their mechanisms of action are different, it is possible that the two compound might have some additive or synergistic effect on aquatic animal production through dietary supplementation with a combination of these compounds. In fact, the health-promoting effect of a dietary mixture of SCFA (formate, propionate, and acetate) and cinnamaldehyde in Pacific white shrimp has been demonstrated in one recent study [32]. The present study was conducted to evaluate the potential benefits of utilizing a blend of short- and medium-chain fatty acid monoglycerides (namely, monobutyrin, monocaprylin, monocaprin, and monolaurin) and cinnamaldehyde (SMMG) as a feed supplement for Pacific white shrimp. The parameters investigated included the growth performance, survival rate, feed conversion ratio (FCR), innate immune function, intestinal *Vibrio* spp. count, and tolerance to hypoxic conditions.

## Materials and methods

### Experimental diets

The commercial product of SMMG (available on the market under the commercial name LipoVital Protect Aqua, Berg &Schmidt Asia Pte Ltd) was used as a feed supplement in the present study. The SMMG used in this study is a blend of α-monoglycerides of butyric acid (C4, monobutyrin or glycerol monobutyrate), caprylic acid (C8, monocaprylin or glycerol monocaprylate), capric acid (C10, monocaprin or glycerol monocaprate), lauric acid (C12, monolaurin or glycerol monolaurate), and cinnamaldehyde (~ 60% total glyceride content). Four experimental diets were formulated with different concentrations of SMMG: 0 (control), 0.3, 0.4, and 0.5% diets. The ingredients and proximate composition of the basal diet are shown in Table 1. To create the experimental diets, SMMG was mixed with other ingredients and then formed into pellets using a pelleting machine (Starmoon, Thailand). All the feeds were kept in the refrigerator at 4°C until use.

**Table 1:** Ingredients and proximate composition (%) of the basal diet.

Ingredients	Percent
Fish meal 60% CP	8.70
Shrimp & squid meal	11.91
Tuna liver powder	5.35
Poultry meal 64% CP	9.04
Soybean meal	15.65
Wheat flour	19.57
Rice bran	1.57
Premix	2.48
Mono calcium phosphate	1.09
Vitamin C 35%	0.09
Carbon	1.52
Calcium carbonate	1.09
Fish & squid soluble	4.35
Lecithin	2.39
Shrimp pellet reprocess	13.04
Fish pellet reprocess	2.17
**Proximate composition (%)**	
Protein (%)	39.88
Lipid (%)	6.80
Crude fiber (%)	3.65
Moisture (%)	8.91
Ash (%)	12.96

### Experiment 1: Effects of SMMG on the growth, survival, and FCR of Pacific white shrimp post-larvae

#### Experimental animals

Two thousand Pacific white shrimp (*Litopenaeus vannamei*) post-larvae 10 (PL-10) were obtained from a commercial shrimp hatchery in Chachoengsao Province. They were transported to the Aquaculture Business Research Center (ABRC) laboratory, Faculty of Fisheries, Kasetsart University, Thailand, and were reared in a 500-L fiberglass tank until reaching PL-12 (about 3 mg/shrimp). Following the acclimation, the shrimp were randomly divided into 24 500 L fiberglass tanks (6 replicates/group). The stocking density was 50 shrimp/tank which was equivalent to 100 shrimp/m^2^. The salinity and temperature of the rearing water were maintained at 25 ppt and 28–30°C, respectively. The water quality parameters, including dissolved oxygen (DO, 5.5–6.5 mg/L, YSI PRO 20, YSI Inc./Xylem Inc., Yellow Springs, OH, USA), pH (8.0–8.3, EcoScan pH 5, Thermo Fisher Scientific Inc.), alkalinity (115–142 mg/L as CaCO_3_), total ammonia (< 0.4 mg/L) and nitrite (< 0.2 mg/L) were analyzed once a week and maintained in an appropriate range for aquaculture of Pacific white shrimp. The alkalinity, total ammonia, and nitrite were analyzed following the methods of the American Public Health Association, American Water Works Association, and Water Environment Federation [33]. During the feeding trial, any leftover feed and feces were siphoned out and about 20% of the rearing water was changed weekly. The animal study was approved by the Kasetsart University Institutional Animal Care and Use Committee (IACUC approval number: ACKU66-FIS-016).

#### Growth and survival study

The feeding trial of the shrimp with SMMG supplementation was conducted for 30 days. During this period, the shrimp in each group were fed to satiation 4 times per day. The body weight, survival rate, and feed conversion ratio (FCR) of shrimp in each group were determined at the end of the feeding trial.

### Experiment 2: Effects of SMMG on the growth, survival, immune responses, intestinal *Vibrio* spp. count, and histological change of Pacific white shrimp under hypoxic stress

#### Experimental animals

The surviving juvenile shrimp from Experiment 1 (approximately 1.5–2 g) were subjected to the hypoxic stress test. Thirty shrimp from each tank of Experiment 1 were randomly re-distributed into new 24 500 L-fiberglass tanks. During the test, the DO was adjusted and maintained at 2–2.5 mg/L for 14 days. Other water parameters, including salinity, temperature, pH, alkalinity, ammonia, and nitrite were maintained in the appropriate range for the Pacific white shrimp as described in Experiment 1. The experimental feed was also given to the shrimp in each group similar to those of Experimental 1. Specific growth rate (SGR) and survival rate were determined at the end of the feeding trial.

#### Immunological study

At the end of the 14 days of the feeding trial under hypoxic conditions, 6 shrimp/group were collected and subjected to the immunological study, including total hemocyte count (THC), phagocytic activity, phenoloxidase (PO) activity, and superoxide dismutase (SOD) activity.

Hemolymph was collected from the ventral sinus of the shrimp using a syringe containing 2 volumes of sodium citrate (10%) as an anticoagulant. Hemocytes from 20 μL of the hemolymph-anticoagulant mixture were counted under a light microscope. The total number of hemocytes was calculated and reported as cells/mL.

Phagocytic activity was determined following the method of Itami et al. [34]. Briefly, the hemocytes were washed and adjusted to 10^5^ cells/mL using shrimp saline. 200 μL of the cell suspension was inoculated onto a cover slip and incubated for 20 min at room temperature. After incubation, the hemocytes were rinsed three times with shrimp saline. Heat-killed yeast (2 mL) was added and incubated for 2 hours at room temperature. After incubation, the cell was rinsed five times with shrimp saline and fixed using methanol. After fixing, the hemocytes were stained with eosin and methylene blue and mounted with Permount Mounting Medium. Then, hemocytes (a total of 200 cells) were counted under the light microscope. The phagocytic activity was calculated and expressed as the percentage of phagocytic hemocytes to the total hemocytes.

PO activity was determined following the method of Söderhäll and Hall [35]. Briefly, 200 μL of supernatant, collected after centrifugation of 500 μL of hemolymph-anticoagulant mixture at 10,000 rpm 4°C for 10 min, was mixed with 200 μL of 0.1% trypsin in cacodylate buffer and incubated for 2–3 min. After incubation, 200 μL of L-dihydroxyphenylalanine (L-DOPA, 4 mg/mL) was added. Then, the enzymatic activity was determined spectrophotometrically at 490 nm using a spectrophotometer. The total protein content was determined following the method described by Lowry et al. [36]. The PO activity was calculated and reported as an increase in optical density per minute per milligram of protein. The SOD activity was determined using a commercial SOD Assay Kit (Sigma-Aldrich, USA) following the protocol of the manufacturer.

#### Intestinal *Vibrio* spp. Count

The same shrimp used for immunological studies were subjected to intestinal *Vibrio* spp. counts. The intestine of each shrimp was collected and weighed. The tissue was homogenized in 1 mL of 1.5% NaCl using a sterile pestle, spread on thiosulfate-citrate-bile salts-sucrose (TCBS) agar, and incubated at 37°C for 18–24 h. After incubation, the colonies were enumerated, and total *Vibrio* spp. was expressed as colony forming unit (CFU)/g.

#### Histopathological study

The histopathological changes of the shrimp’s hepatopancreas after the 14-day-hypoxic condition were examined following the method of Bell and Lightner [37]. Six shrimp from each group were randomly collected and fixed with Davidson’s fixative for 48 h. After fixing, the shrimp were kept in 70% ethanol until use for histopathological study.

### Statistical analysis

IBM SPSS ver. 27 (IBM Corporation, Armonk, NY, USA) was used for statistical analysis. One-way analysis of variance (ANOVA) and Duncan’s multiple range test were used to test the difference among the mean values of each treatment group. The values were considered statistically different when *p*-value was less than 0.05.

## Results

### Experiment 1: Effects of SMMG on the growth performance, survival, and FCR of Pacific white shrimp post-larvae

The final body weight, survival rate, and FCR of shrimp after being fed with different concentrations of SMMG for 30 days are shown in Table 2. The average final body weights of the 0.3–0.5% SMMG groups were in the range of 2.81–3.06 g, which were significantly higher than the control shrimp (1.96 g). The highest survival rate was seen in the 0.5% SMMG-fed shrimp (84.33%), followed by the 0.4 and 0.3% SMMG groups, which were 77.00 and 74.00%, respectively; the survival rates of all SMMG groups were significantly greater than the control (68.33%). Regarding the FCR, even though the 0.5% SMMG group showed the best FCR at 1.38, there were no statistically significant differences compared to the other groups (1.40–1.53).

**Table 2:** The final body weight, survival rate, and feed conversion ratio (FCR) of Pacific white shrimp post-larvae following the 30 day-feeding trial (Experiment 1).

Treatment groups	Final body weight (g)	Survival rate (%)	FCR
Control	1.96±0.35^b^	68.33±4.46^c^	1.53±0.20^a^
0.3% SMMG	2.81±0.49^a^	74.00±3.57^b^	1.50±0.39^a^
0.4% SMMG	2.99±0.24^a^	77.00±4.33^b^	1.40±0.15^a^
0.5% SMMG	3.06±0.38^a^	84.33±4.08^a^	1.38±0.21^a^

Data are presented as mean ± standard deviation. Means in the same column with different superscript are significantly different from each other (p < 0.05).

### Experiment 2: Effects of SMMG on the growth, survival, immune responses, intestinal *Vibrio* spp. count, and histological change of Pacific white shrimp under hypoxic stress

The SGR, survival rate, and intestinal *Vibrio* spp. count of shrimp fed with different levels of SMMG under the hypoxic stress conditions for 14 days are presented in Table 3. It was demonstrated that the average SGR of shrimp, which ranged from 3.31–3.96%/day, were not significantly affected by the SMMG supplementations under the low DO conditions. However, the survival rate was significantly increased when SMMG was added to the diets; the highest survival was observed in the 0.5% SMMG group (80.00%), whereas the lowest one was seen in the control group (51.67%). Likewise, the 0.5% SMMG shrimp had the lowest number of *Vibrio* spp. in the intestine (2.68 x 10^4^ CFU/g), while the control shrimp exhibited the highest *Vibrio* spp. count (4.68 x 10^4^ CFU/g).

**Table 3:** The specific growth rate (SGR), survival rate, and intestinal *Vibrio* spp. count of Pacific white shrimp under the hypoxic stress test for 14 days (Experiment 2).

Treatment groups	SGR (%/day)	Survival rate (%)	Intestinal *Vibrio* spp. count (10^4^ CFU/g)
Control	3.96±1.34^a^	51.67±5.05^d^	4.68±0.15^c^
0.3% SMMG	3.31±1.08^a^	61.11±3.44^c^	3.50±0.04^c^
0.4% SMMG	3.43±1.04^a^	71.67±4.60^b^	3.06±0.02^b^
0.5% SMMG	3.57±0.72^a^	80.00±3.65^a^	2.68±0.41^a^

Data are presented as mean ± standard deviation. Means in the same column with different superscript are significantly different from each other (p < 0.05).

**Fig 1:**
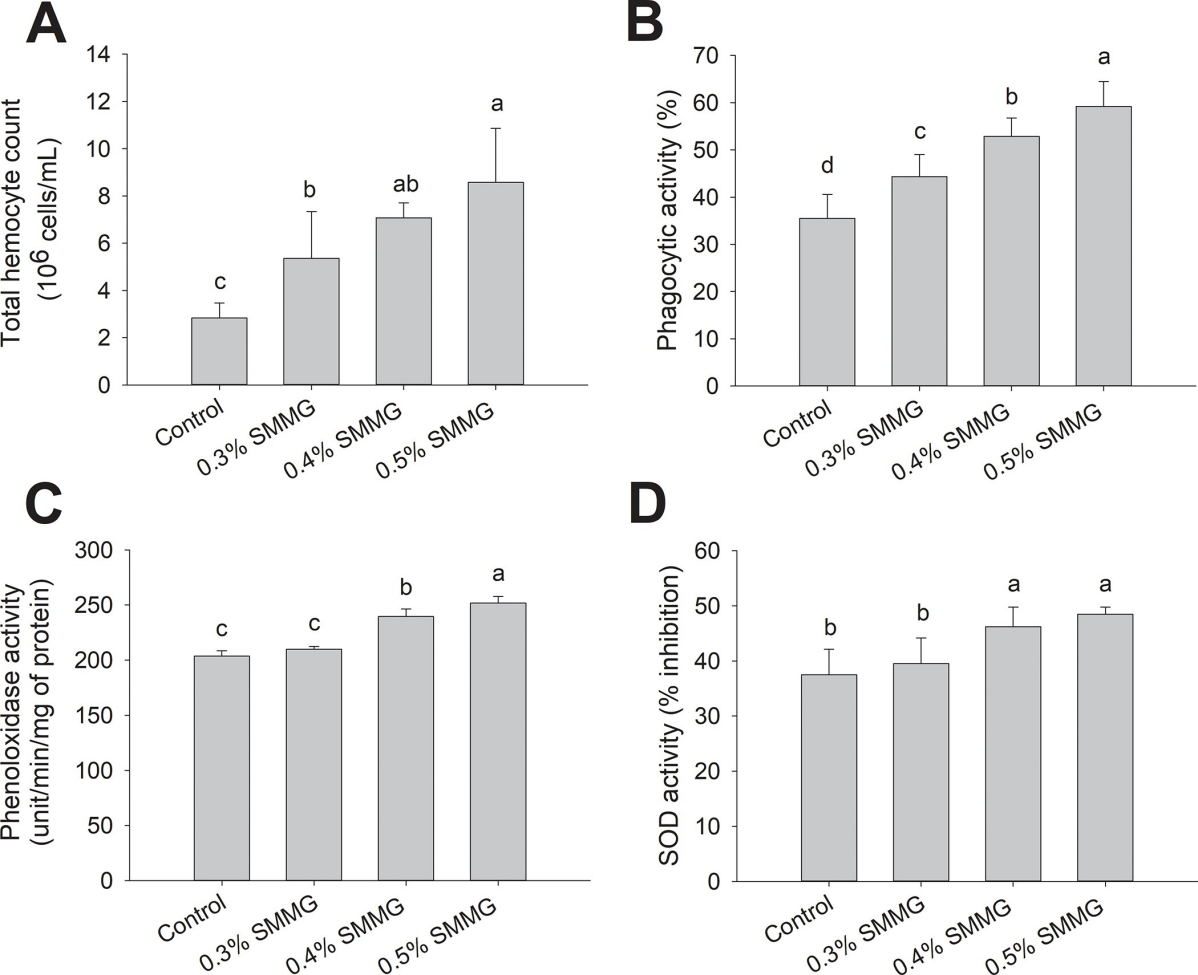
Immune parameters of Pacific white shrimp under the hypoxic stress test for 14 days. Total hemocyte count (10^6^ cells/mL) (A), phagocytic activity (%) (B), phenoloxidase activity (unit/min/mg of protein) (C), and superoxide dismutase (SOD) activity (% inhibition), (D) of the shrimp in the control, 0.3% SMMG, 0.4% SMMG, and 0.5% SMMG groups. The data are presented as the mean ± standard deviation. Different letters above the bars indicate significant differences (p < 0.05).

The immunostimulatory activity of SMMG under the low DO conditions was evident as shown in Fig 1. The average THC of the 0.4–0.5% SMMG-fed shrimp was 7.08–8.58 x 10^6^ CFU/mL, which was significantly higher than the control (2.83 x 10^6^ CFU/mL). Similarly, significant improvements in the phagocytic, PO, and SOD activities of the 0.4–0.5% SMMG groups (which were 53–59%, 240–252 units/min/mg protein, and 46–48%, respectively) were also observed compared to the control (which were 36%, 204 units/min/mg protein, and 37%, respectively).

**Fig 2:**
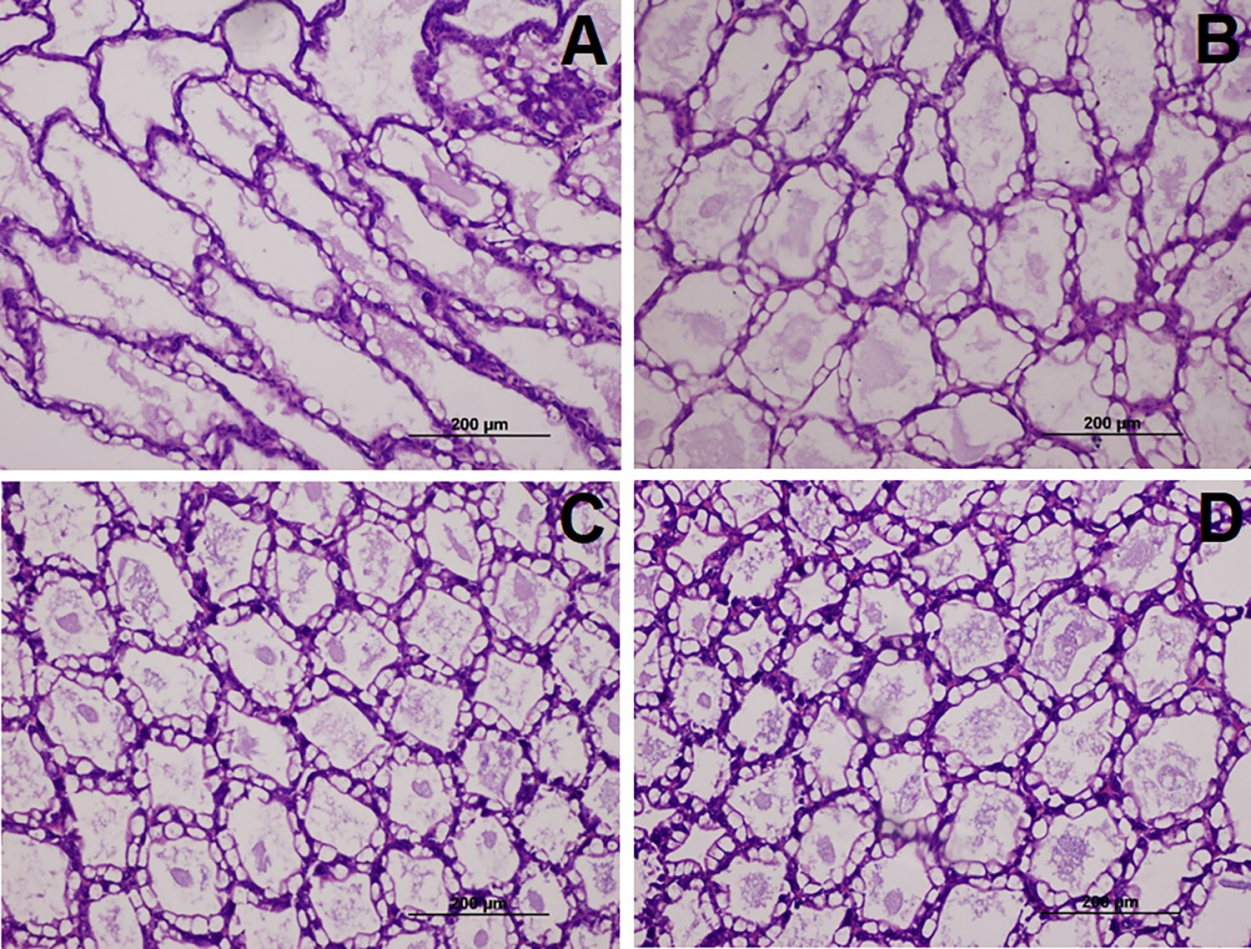
Histopathology of the hepatopancreas of Pacific white shrimp under the hypoxic stress test for 14 days: (A) control, (B) 0.3% SMMG, (C) 0.4% SMMG, and (D) 0.5% SMMG groups (H&E stain).

Regarding histopathological changes in the hepatopancreas, the control and 0.3% SMMG-fed shrimp subjected to low DO challenge revealed signs of less lipid accumulation and epithelial cell atrophy (Fig 2). On the other hand, no histopathological changes were observed in the 0.4–0.5% SMMG groups.

## Discussion

Monoglycerides of SCFA, like monobutyrin [38], and MCFA, such as monolaurin [20,21], mixtures of SCFA and MCFA monoglycerides [22,24], cinnamaldehyde [29–31], as well as SCFA plus cinnamaldehyde [32] have been demonstrated to effectively enhance the growth performance of Pacific white shrimp. Similar outcomes were observed in the current study, which employed SMMG (a combination of monobutyrin, monocaprylin, monocaprin, monolaurin, and cinnamaldehyde) at concentrations of 0.3–0.5% diet. It is noteworthy that a blend of MCFA consisting of caproic acid, caprylic acid, capric acid, and lauric acid at concentrations up to 0.5% was unable to enhance the body weight of shrimp even if the immune function and resistance to *V*. *parahaemolyticus* infection showed a significant improvement [39]. It is believed that the growth-enhancing effect might be associated with the antimicrobial action of monoglycerides [12,14,15] and cinnamaldehyde [40–42] that suppress the growth of pathogenic bacteria and subclinical infections, enabling more energy to be available for growth. Nevertheless, additional experiments are needed to demonstrate the correlation between the growth performance and antimicrobial efficacy of monoglycerides. Additionally, previous studies have shown that adding either monoglycerides or cinnamaldehyde to the diet increased digestive enzyme activities in shrimp [20,21,29] and fish [18,19,27], potentially contributing to their growth-promoting effects.

The immunomodulatory activity of MCFA and their monoglyceride has been discovered although the exact mechanism has yet to be disclosed [8,13]. In our study, the immune parameters including THC, phagocytic, PO, and SOD activities were increased in the 0.4–0.5% SMMG groups. The immunostimulatory results were also observed in shrimp fed short‑chain and medium‑chain fatty acid glycerides (0.035–0.11% diet) and monolaurin (0.035–0.07% diet) which showed upregulation of prophenoloxidase (proPO) gene expression [20,24]. Likewise, dietary supplementation with monolaurin increased white blood cell count, IgM, and IgG in the serum of Nile tilapia (*Oreochromis niloticus*) [18]. Less information is known regarding the immunomodulatory property of cinnamaldehyde compared to that of glycerides. Nevertheless, it was found that the immune-related genes of zebrafish (*Danio rerio*) [43] and striped snakehead (*Channa striata*) [44] were significantly elevated by cinnamaldehyde.

Although dietary monoglycerides and cinnamaldehyde were reported to enhance disease resistance against vibriosis in fish [16,19,43] and shrimp [22,24,29], to the best of the authors’ knowledge the effect of monoglycerides in improving tolerance against hypoxic conditions has not been evaluated so far. Our findings showed that the survival rates of shrimp fed 0.3–0.5% SMMG-supplemented diets were significantly higher than the control under the hypoxic stress conditions, suggesting an increase in hypoxic tolerance. Hypoxic conditions can sometimes be found in shrimp ponds in the cases of insufficient aeration, massive phytoplankton die-off, or excessive organic waste accumulation in the ponds. The low DO level is associated with oxidative stress [45] which leads to impaired growth performance, immune function, and disease resistance [46–48]. The enhanced hypoxic stress tolerance observed in the SMMG-fed shrimp might be partially attributed to the improved antioxidant capacity as indicated by increased activities of antioxidant enzymes such as superoxide dismutase (SOD), catalase (CAT), and glutathione peroxidase (GPx), as well as decreased the level of malondialdehyde (MDA), a marker of oxidative stress, in the previous studies [16,18,21,24,28,29,32].

The modulation of gut microbiota is also believed to play a role in the beneficial effects of SMMG, including the enhancement of shrimp’s growth performance and tolerance to hypoxic stress. In our case, the total number of *Vibrio* spp., a common opportunistic pathogen of marine shrimp, in the intestine was significantly reduced in the shrimp fed 0.4–0.5% SMMG, presumably due to the antimicrobial activity of monoglycerides and cinnamaldehyde. A similar observation was also seen in the previous work using 0.6–0.8% monoglycerides (a mixture of monobutyrin, monocaprylin, and monocaprin) which exhibited a significant decrease in the total *Vibrio* spp. count in the hepatopancreas [22]. MCFA like caprylic acid, capric acid, and lauric acid completely inhibited the growth of *V*. *anguillarum* and *V*. *harveyi* at the concentrations of 271–751 μg/mL (1.88–3.75 mM) and 1,082–1,502 μg/mL (7.5 mM), respectively [49]. Monoglyceride of caprylic acid (i.e., monocaprylin) was more effective against fish pathogens such as *Edwardsiella ictaluri*, *E*. *tarda*, *Streptococcus iniae*, and *Yersinia ruckeri* than caprylic acid, with the minimum inhibitory concentration (MIC) between 546–1,092 μg/mL (2.5–5 mM) and 1,082–1,442 μg/mL (7.5–10 mM), respectively [14]. According to the literature data, the antibacterial action of cinnamaldehyde is generally more potent than that of monoglycerides, with the MIC of 10–150 μg/mL against several fish pathogens including *Vibrio harveyi*, *Aeromonas hydrophila*, *A*. *salmonicida* sub sp. *salmonicida*, *E*. *ictaluri*, *E*. *tarda*, *S*. *iniae*, and *Flavobacterium columnare* [40–42]. Moreover, supplementation of either monoglycerides or cinnamaldehyde in aquafeed has been shown to improve balancing between beneficial and pathogenic bacteria in the gut of aquatic animals. Gut microbiome study in hybrid grouper (*Epinephelus fuscoguttatus* × *E*. *lanceolatus*) fed 0.18% monolaurin not only exhibited a significant reduction in the relative abundance of *Vibrio* spp. but also increasing the proportion of *Bacillus* spp. [16]. Similarly, feeding gilthead sea bream (*Sparus aurata*) with 0.5% monoglycerides (a mixture of monopropionin, monobutyrin, monocaproin, monoheptanoin, monocaprylin, and monolaurin) led to a decrease in the relative abundance of bacteria in Vibrionaceae family in the gut, and increase the occurrence of Lactobacillaceae family [23]. Lastly, dietary supplementation of 0.1% cinnamaldehyde in the feed of tongue sole (*Cynoglossus semilaevis*) resulted in an increase in the abundant of potential probiotic bacteria *Bacillus*, *Lactobacillus*, and *Bifidobacterium* in the gut [28].

It was noted that SMMG at a level of 0.3% diet could not reduce the number of *Vibrio* spp. count in the intestine of the shrimp in this study, but the survival rate of the shrimp was significantly enhanced. This effect might be due to the ability of medium‑chain fatty acid glycerides, especially monolaurin, to inhibit bacterial toxin production at a concentration below growth inhibition [50–52]. This was due to the interference of signal transduction that reduced virulent factor expression of bacteria [51,53]. Although these mechanisms have been well-documented using monolaurin against Gram-positive bacteria, including *Staphylococcus aureus*, there have been no reports against *Vibrio* spp., and it is worth investigating in future studies.

## Conclusions

Feeding the Pacific white shrimp with SMMG at 0.4–0.5% diet for 30 days resulted in a significant increase in body weight and survival rate compared to the control shrimp. The shrimp also showed better tolerance to hypoxic stress conditions, as well as improved immune parameters, reduced intestinal *Vibrio* spp. count, and improved hepatopancreas conditions. This study highlights the benefits of using a short- and medium-chain fatty acid monoglyceride and cinnamaldehyde mixture as a feed additive in shrimp aquaculture. Future study is required to elucidate the mechanisms of monoglycerides and cinnamaldehyde that are responsible for health promotion, especially in improving immune response and hypoxic tolerance of shrimp.

## Supporting information

S1 Table The final body weight, survival rate, and feed conversion ratio (FCR) of Pacific white shrimp post-larvae following the 30 day-feeding trial (Experiment 1). (DOCX)

S2 Table Immune parameters of Pacific white shrimp under the hypoxic stress test for 14 days (Experiment 2). (DOCX)

S3 Table The specific growth rate (SGR), survival rate, and intestinal *Vibrio* spp. count of Pacific white shrimp under the hypoxic stress test for 14 days (Experiment 2). (DOCX)
