# Supplementary material for: Effects of monoglycerides of short and medium chain fatty acids and cinnamaldehyde blend on the growth, survival, immune responses, and tolerance to hypoxic stress of Pacific white shrimp (Litopenaeus vannamei)
Source: PLoS One. 2024 Aug 8;19(8):e0308559. doi: 10.1371/journal.pone.0308559 (PMC11309431; doi:10.1371/journal.pone.0308559)
Supplement: S1 Table — (DOCX) [file pone.0308559.s001.docx]

**S1 Table. The final body weight, survival rate, and feed conversion ratio (FCR) of Pacific white shrimp post-larvae following the 30 day-feeding trial (Experiment 1).**

| **Treatment** | **Final body weight (g)** | | **Survival rate (%)** | | **FCR** | |
| --- | --- | --- | --- | --- | --- | --- |
|  | **Raw data** | **mean ± SD** | **Raw data** | **mean ± SD** | **Raw data** | **mean ± SD** |
| **Control 1** | 2.46 | 1.96±0.35 | 64 | 68.33±4.46 | 1.27 | 1.53±0.20 |
| **Control 2** | 1.58 |  | 76 |  | 1.67 |  |
| **Control 3** | 2.29 |  | 68 |  | 1.28 |  |
| **Control 4** | 1.70 |  | 68 |  | 1.73 |  |
| **Control 5** | 1.75 |  | 70 |  | 1.63 |  |
| **Control 6** | 1.95 |  | 64 |  | 1.60 |  |
| **0.3% SMMG 1** | 1.90 | 2.81±0.49 | 70 | 74.00±3.57 | 2.26 | 1.50±0.39 |
| **0.3% SMMG 2** | 3.12 |  | 76 |  | 1.27 |  |
| **0.3% SMMG 3** | 2.85 |  | 72 |  | 1.46 |  |
| **0.3% SMMG 4** | 3.18 |  | 72 |  | 1.31 |  |
| **0.3% SMMG 5** | 3.17 |  | 80 |  | 1.18 |  |
| **0.3% SMMG 6** | 2.66 |  | 74 |  | 1.52 |  |
| **0.4% SMMG 1** | 3.16 | 2.99±0.24 | 80 | 77.00±4.33 | 1.27 | 1.40±0.15 |
| **0.4% SMMG 2** | 2.68 |  | 72 |  | 1.66 |  |
| **0.4% SMMG 3** | 2.99 |  | 76 |  | 1.41 |  |
| **0.4% SMMG 4** | 3.18 |  | 80 |  | 1.26 |  |
| **0.4% SMMG 5** | 3.23 |  | 72 |  | 1.38 |  |
| **0.4% SMMG 6** | 2.72 |  | 82 |  | 1.43 |  |
| **0.5% SMMG 1** | 3.19 | 3.06±0.38 | 84 | 84.33±4.08 | 1.31 | 1.38±0.21 |
| **0.5% SMMG 2** | 2.45 |  | 80 |  | 1.79 |  |
| **0.5% SMMG 3** | 3.50 |  | 80 |  | 1.25 |  |
| **0.5% SMMG 4** | 3.01 |  | 84 |  | 1.38 |  |
| **0.5% SMMG 5** | 3.37 |  | 88 |  | 1.18 |  |
| **0.5% SMMG 6** | 2.83 |  | 90 |  | 1.37 |  |
