# Supplementary material for: Effects of monoglycerides of short and medium chain fatty acids and cinnamaldehyde blend on the growth, survival, immune responses, and tolerance to hypoxic stress of Pacific white shrimp (Litopenaeus vannamei)
Source: PLoS One. 2024 Aug 8;19(8):e0308559. doi: 10.1371/journal.pone.0308559 (PMC11309431; doi:10.1371/journal.pone.0308559)
Supplement: S2 Table — (DOCX) [file pone.0308559.s002.docx]

**S2 Table. Immune parameters of Pacific white shrimp under the hypoxic stress test for 14 days (Experiment 2).**

| **Treatment** | **Total hemocyte count (10^6^ cells/mL)** | | **Phagocytic activity (%)** | | **Phenoloxidase activity (unit/min/mg of protein)** | | **SOD activity (% inhibition)** | |
| --- | --- | --- | --- | --- | --- | --- | --- | --- |
|  | **Raw data** | **mean ± SD** | **Raw data** | **mean ± SD** | **Raw data** | **mean ± SD** | **Raw data** | **mean ± SD** |
| **Control 1** | 3.30 | 2.83±0.64 | 31 | 35.50±5.09 | 206.79 | 203.57±4.75 | 34.80 | 37.47±4.66 |
| **Control 2** | 2.85 |  | 34 |  | 198.93 |  | 32.71 |  |
| **Control 3** | 2.40 |  | 29 |  | 208.93 |  | 41.76 |  |
| **Control 4** | 2.10 |  | 40 |  | 206.07 |  | 41.07 |  |
| **Control 5** | 2.48 |  | 42 |  | 203.93 |  | 32.37 |  |
| **Control 6** | 3.83 |  | 37 |  | 196.79 |  | 42.11 |  |
| **0.3% SMMG 1** | 2.78 | 5.36±1.98 | 38 | 44.33±4.68 | 213.21 | 209.88±2.62 | 41.76 | 39.50±4.68 |
| **0.3% SMMG 2** | 3.00 |  | 42 |  | 208.21 |  | 35.15 |  |
| **0.3% SMMG 3** | 6.45 |  | 43 |  | 208.93 |  | 42.11 |  |
| **0.3% SMMG 4** | 7.43 |  | 52 |  | 208.21 |  | 46.29 |  |
| **0.3% SMMG 5** | 6.68 |  | 45 |  | 207.50 |  | 34.11 |  |
| **0.3% SMMG 6** | 5.85 |  | 46 |  | 213.21 |  | 37.59 |  |
| **0.4% SMMG 1** | 7.73 | 7.08±0.63 | 48 | 52.83±3.87 | 236.79 | 239.52±6.90 | 51.51 | 46.23±3.52 |
| **0.4% SMMG 2** | 7.58 |  | 59 |  | 230.36 |  | 45.24 |  |
| **0.4% SMMG 3** | 7.20 |  | 55 |  | 244.64 |  | 46.64 |  |
| **0.4% SMMG 4** | 6.30 |  | 52 |  | 235.36 |  | 45.59 |  |
| **0.4% SMMG 5** | 6.30 |  | 50 |  | 249.64 |  | 47.68 |  |
| **0.4% SMMG 6** | 7.35 |  | 53 |  | 240.36 |  | 40.72 |  |
| **0.5% SMMG 1** | 5.48 | 8.58±2.28 | 54 | 59.17±5.27 | 255.36 | 251.79±5.98 | 50.12 | 48.49±1.28 |
| **0.5% SMMG 2** | 6.53 |  | 54 |  | 255.36 |  | 48.72 |  |
| **0.5% SMMG 3** | 10.20 |  | 65 |  | 255.36 |  | 46.98 |  |
| **0.5% SMMG 4** | 7.88 |  | 57 |  | 248.21 |  | 47.33 |  |
| **0.5% SMMG 5** | 10.58 |  | 59 |  | 255.36 |  | 48.03 |  |
| **0.5% SMMG 6** | 10.80 |  | 66 |  | 241.07 |  | 49.77 |  |
